# Gut Immunobiosis and Biomodulators

**DOI:** 10.3390/nu15092114

**Published:** 2023-04-28

**Authors:** Vito Leonardo Miniello, Andrea Miniello, Laura Ficele, Aleksandra Skublewska-D’Elia, Vanessa Nadia Dargenio, Fernanda Cristofori, Ruggiero Francavilla

**Affiliations:** 1 Nutrition Unit, Department of Pediatrics, “Giovanni XXIII” Children Hospital, University of Bari Aldo Moro, 70126 Bari, Italy; vito.miniello@libero.it (V.L.M.);; 2 Department of Emergency and Organ Transplantation, School of Allergology and Clinical Immunology, University of Bari Aldo Moro, Policlinico di Bari, 70126 Bari, Italy; 3 Interdisciplinary Department of Medicine, Pediatric Section, Children’s Hospital ‘Giovanni XXIII’, University of Bari Aldo Moro, 70126 Bari, Italyrfrancavilla@gmail.com (R.F.)

**Keywords:** gut microbiota, eubiosis, dysbiosis, biomodulators, prebiotics, probiotics, infant formulas, food allergy

## Abstract

The human gastrointestinal (GI) tract hosts complex and dynamic populations of microorganisms (gut microbiota) in advantageous symbiosis with the host organism through sophisticated molecular cross-talk. The balance and diversification within microbial communities (eubiosis) are crucial for the immune and metabolic homeostasis of the host, as well as for inhibiting pathogen penetration. In contrast, compositional dysregulation of the microbiota (dysbiosis) is blamed for the determinism of numerous diseases. Although further advances in the so-called ‘omics’ disciplines are needed, dietary manipulation of the gut microbial ecosystem through biomodulators (prebiotics, probiotics, symbionts, and postbiotics) represents an intriguing target to stabilize and/or restore eubiosis. Recently, new approaches have been developed for the production of infant formulas supplemented with prebiotics (human milk oligosaccharides [HMOs], galacto-oligosaccharides [GOS], fructo-oligosaccharides [FOS]), probiotics, and postbiotics to obtain formulas that are nutritionally and biologically equivalent to human milk (closer to the reference).

## 1. Introduction

### 1.1. One, None, a Hundred Thousand…Self

In the last two decades, complex systems biology has been developed [1]. This discipline investigates collective properties that cannot be connoted by analyzing a single component but rather by examining the complex totality through a multidisciplinary approach. The human body is a sophisticated multisystem regulated by genetic background, epigenetics, environmental drivers, the microbiome, interpersonal relationships, activities, emotions, and experience. The holistic version (from Greek όλος: totality) considers our organism in its entirety (body, mind, experience and mood) and not as a collection of organs and systems. The human gastrointestinal (GI) tract hosts one of the most complex ecosystems on the planet. The vast community of resident microbes represents a complex system interconnected with the host organism (co-evolved over time) and regulated by sophisticated and vulnerable dynamics. The actual self is no longer a distinguishing feature of the human being but an advantageous structural and functional combination with that microscopic universe we used to call ‘non-self.’ Through vaginal birth, an advantageous ‘handover’ occurs from mother to infant (‘microbial inheritance’). During and immediately after birth, a significant number of maternal and environmental microorganisms colonize the skin and mucous membranes (oral cavity, airways, urogenitals and GI tracts). As a result of the post-natal colonization process, site-specific microbial ecosystems, known as microbiota, are formed. The microbiome, on the other hand, includes the microbiota, consisting predominantly of bacteria (although other domains such as archaea, fungi, and algae are also present), and their microbial structural elements (proteins/peptides, lipids, polysaccharides), nucleic acids (structural DNA/RNA), mobile genetic elements (viruses, phages, residual DNA), and microbial metabolites (signal molecules, toxins, organic, and inorganic compounds) [2]. This multitude of microorganisms becomes an integral part of the host (self). Thus, the human body is legitimately considered a holobiont (‘superorganism’) consisting of its own eukaryotic cells and various microbiomes. The term ‘homo bacteriens,’ coined by Henderson and Wilson, renders the concept of mutualism more accurate than others [3].

### 1.2. In Gut We Trust

The human GI tract has been perceived as an organ exclusively dedicated to digestive functions for a long time. This conception has been radically overturned and integrated over the last few decades, when it became apparent that the microbial biomass performs regulatory processes with local and systemic effects, as well as a noticeable impact on metabolism, immunity, behavior, mood, and local and systemic inflammation [4]. Under eubiotic conditions, the gut microbiota remotely regulates the functions of various organs and systems. A ‘healthy’ microbiota is characterized by ecological stability and resilience (ability to resist changes in the microbial community under stress or to restore its composition), by the presence of specific bacterial patterns (likely associated with health), or by beneficial functional profiles induced by our commensals (trophic, metabolic, immune, and protective) [5]. 

Host/microbiome symbiosis occurs through interactions within microorganisms and between microbiota and host. The concept of ‘core microbiome’ (i.e., a common set of microbial populations that is shared across all individuals) has long been a matter of study, but the scientific community still struggles in the identification of the stable components that make up this core. Recently, Sharon et al. conducted a review of the literature available on the concept, highlighting the current one-dimensional approach of the research on the subject (which mostly focuses on genomic and taxonomic classification) and emphasizing that the core microbiome needs to be considered in the context of the diet, geography, age, and health state of the host [6]. 

It is estimated that the gut microbial genome consists of around three million genes, an order of magnitude of 150 times more than that of humans, even considering the massive presence of viruses (collectively referred to as virome) with higher percentages than the bacteria themselves (ratio of 1:1 to 10:1). Our organism produces few gastrointestinal enzymes. In contrast, microbial biomass provides hundreds of them (complementary and specific) that are essential in numerous metabolic processes. Thus, the metabolic heritage of the gut microbiome extends our biochemical flexibility by providing a valuable repertoire of enzymes not encoded by the human genome and involved in tasks such as the synthesis of vitamins and polyphenols and the digestion of polysaccharides. This prerogative is believed to be the outcome of evolutionary pressure that led bacteria to become symbionts.

Several scientific shreds of evidence reinforce the concept of the gut microbiota as a ‘bacterial organ’ with useful local and systemic functions [7] (Table 1).

The composition of gut microbiota in the early months of life is significantly influenced by numerous intrinsic and extrinsic factors such as genetic background, mode of delivery (vaginal or caesarean), antibiotic therapy in the perinatal period; gestational age and APGAR score (score calculated on a newborn at 1 and 5 min after birth evaluating breathing effort, heart rate, muscle tone, reflexes and skin color), delivery site (nosocomial or home), mode of feeding (maternal, artificial or mixed), complementary feeding (timing, composition), breastmilk oligosaccharide pattern (presence or absence of breast milk secretor and/or Lewis status), atopy, body mass index, maternal weight gain during pregnancy, and pet keeping [8]. 

The host organism influences the composition of the microbiota by producing specific [microRNAs (miRNAs)] and non-specific factors [antimicrobial peptides, mucus class A immunoglobulins (IgA)] that promote the growth of specific bacterial genera while inhibiting that of others. 

MicroRNAs are the most characterized class of non-coding RNAs (ncRNA). Recent emerging evidence has revealed that ncRNAs [e.g., miRNAs, long non-coding RNAs (lncRNA) or small interfering RNAs (siRNA), circular RNAs] modulate multiple functions of enterocytes and intestinal microbiota as well as host–microbial interactions. Thus, they play a key role as epigenetic drivers and as potential biomarkers of the host response to microbiome-associated pathologies. miRNAs are mainly expressed in cells/tissues, but some of them are secreted by cells in extracellular vesicles or exosomes and circulate in body fluids. Exosomal ncRNAs present in food (exogenous ncRNAs) have been subject of recent interest due to their potential impact on the gut microbiome eubiosis and health.

Regulation of miRNA expression is considered one of the crucial factors for both gut homeostasis and pathological conditions [9,10]. However, miRNAs do not code for any proteins; instead, both endogenous and exogenous (food-derived) miRNAs play a key role in regulating bacterial gene expression, the epithelial barrier (tight junctions), apoptosis, proliferation, and differentiation of enterocytes. In fact, exosomal miRNAs present in food are highly stable, and upon ingestion they can easily reach the gut lumen and affect the microbiome and host gene expression in the intestine [11,12]. 

## 2. Immunobiosis

The gut microbiota plays an essential role in modulating and consolidating the immune system (immuno-modulation). An eubiotic (rich and diverse) microbial ecosystem interacts with the enterocyte and the underlying mucosal gut-associated lymphoid tissues (GALT), activating a sophisticated network in which the two arms of immunity (innate and adaptive) play a leading role. The adaptive immune response takes much longer than the innate compartment, but it is antigen-specific and uses immunological memory to optimize the reaction to a subsequent re-exposure. However, innate immunity is not as memory-less as stigmatized until a few years ago. This theory has recently been revolutionized by Mihai Netea et al. [13], who coined the term ‘trained immunity’ to refer to the increased effectiveness of the innate immune system in counteracting pathogens after an initial challenge, such as vaccination (tuberculosis BCG vaccine) and/or infection. Some pathogen-associated molecular profiles (PAMPs, pathogen-associated molecular patterns) induce lasting epigenetic modifications and metabolic reprogramming by recognizing and binding to specific receptors (PRRs, pattern recognition receptors). These advantageous adaptations result in a more effective response following secondary stimulation with the same or a different ligand.

Considering that the gut microbial biomass carries indisputable benefits and potential aggressiveness, the immune system must discriminate ‘useful’ antigens (i.e., food and commensals) from pathogens or potentially pathogens (pathobionts) to induce tolerance or activation of the immune response, respectively. Since the intestinal epithelium is a front designed to repel pathogens, the host must rely on non specific host-defense mechanisms for barriers of innate immunity (anatomic, physiologic, phagocytic/endocytic and inflammatory). The physical barrier comprises an outer layer of mucus colonized by microorganisms and an inner layer reinforced by inter-epithelial junctions (occluding, communicating, and adherent or anchoring). In the outer layer, the communication process between bacteria (quorum sensing) induces biofilm formation, the production of secondary metabolites, and a bacterial competition system in both Gram-positive and -negative bacteria [14]. 

The biochemical barrier is provided by secretory IgAs (sIgA) and antimicrobial peptides (AMPs), such as α-defensins, bacteriocins, lysozymes, Reg3 proteins, and C-type lectins [15]. Among these, the role of Reg3 proteins in limiting tissue damage and optimizing the related repair processes has recently aroused scientific interest. Recent evidence shows that their function in the intestine is not relegated exclusively to mucosal protection against pathogens, and instead they also induce an advantageous increase in lactobacilli and a reduction in bacterial translocation responsible for inflammation [16]. 

Molecular profiles associated with commensals or pathogens, respectively, MAMPs (microbe-associated molecular patterns) and PAMPs, are molecular combinations that are phylogenetically conserved in the microbial galaxy but not expressed by the host cells and must therefore be sampled regularly by the immune system. Damage-associated molecular patterns (DAMPs) are also recognized and processed in order to obtain a sense of what is happening in the gut habitat. The recognition of these molecular patterns is delegated to transmembrane and intracytoplasmic PRRs. Among these, the Toll-like receptors (TLRs) should be mentioned for their high functional value. TLRs are strategically distributed on epithelial cells and antigen-presenting cells (APCs), such as dendritic cells, B lymphocytes, macrophages, and monocytes. Toll-like receptors are responsible for the recognition of a wide variety of molecules expressed by pathogens but not by host cells (Table 2).

Dendritic cells can either internalize the antigen and process it for presentation to T lymphocytes or keep it on the surface in its native form to make it available to antigen-specific B lymphocytes. The tolerance or eventual reactive response depends on the type of activated receptor (signaling). Each component of the symbiont microbiota is useful for tolerogenesis and the consolidation of the mucosal barrier, which is an advantageous anatomical-functional prerogative aimed at regulating antigen traffic. Commensal structures (DNA, lipoteichoic acid, lipopolysaccharides, MAMPs) and bacterial metabolites (short-chain fatty acids) ensure tolerance through non-immune (epithelial barrier integrity, mucus production, reduced intestinal permeability) and immune action (production of sIgA, anti-inflammatory cytokines and chemokines, induction of tolerogenic dendritic cells, differentiation, and proliferation of regulatory T lymphocytes).

**Figure 1:**
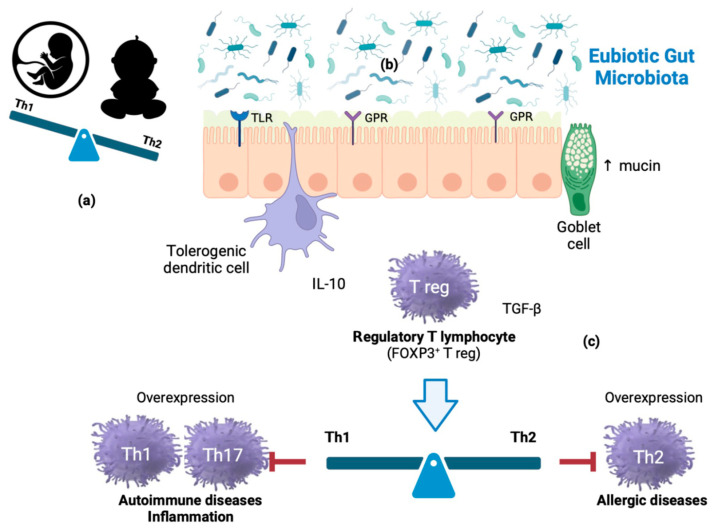
Mechanisms of immune homeostasis modulation: (**a**) During intrauterine life, the fetus represents an antigenic non-self for the maternal immune system. This outcome does not occur due to the fetus peculiar prevalence of Th-2-type immune responses. After birth, however, the Th2-polarized cytokine milieu is inadequate to counter some infections. (**b**) In the first months of life, the antigenic ‘pressure’ provided by a highly diversified, eubiotic microbial biomass would play a decisive role in training the immune system. (**c**) Principal players in immune homeostasis are regulatory T-lymphocytes (Treg), activated by dendritic cells through commensal components and metabolites acting as ligands of receptors such as Toll-like receptors (TLR1 and TLR2) and G- protein coupled receptors (GPR41, GPR43, GPR109), respectively. Th1 cells generate IFN-γ and are involved in cell-mediated immunity; Th2 cells produce IL-4 and contribute to humoral immunity; IL-17-producing Th17 cells play a strategic role in immune responses to extracellular pathogens and fungi. However, Th subset continuous overexpression is involved in autoimmune, inflammatory, and allergic diseases.

During intrauterine life, the product of conception (endowed with a genetic make-up partially inherited from the father) represents an antigenic non-self for the maternal immune system and, as such, is potentially at risk of rejection (abortion). This outcome, physiologically mediated by T helper (Th)-1 lymphocytes, is avoided by the fetus’ peculiar prevalence of Th-2-type immune responses. After birth, however, the Th2-polarized cytokine milieu is inadequate to counter infections; in fact, Th-2 cells confer protection against extracellular pathogens (parasites and bacteria), while Th-1 cells are specialized in protection against intracellular pathogens (viruses and bacteria). Therefore, a beneficial immune conversion (shift) process begins in the first months of life and is completed in the first 3–4 years. The reactive Th-2 state (characteristic of atopic individuals, but physiological in fetal life and early childhood) gradually translates into a condition dominated by Th-1 responses. Atopic individuals retain an ‘immature’ (Th-2) immune system, likely due to an ineffective Th-2→Th-1 shift and/or the deficit of cytokines that catalyze it [e.g., interferon γ (IFN-γ)]. In the first months of life, the antigenic ‘pressure’ provided by a highly diversified, eubiotic microbial biomass would play a decisive role in training the immune system, far more effective than that attributed to fecal-oral infections by the ‘hygiene theory’ postulated in 1989 by the British epidemiologist David Strachan. Principal players in immune homeostasis are regulatory T-lymphocytes (Treg), activated by dendritic cells through compounds and metabolites from commensals that act as ligands of receptors, such as Toll-like receptors (TLR1, TLR2) and G protein-coupled receptors (GPR41, GPR43, and GPR109), respectively (Figure 1).

## 3. Biomodulators and Immuno-Nutrition

The diversification and balance of intestinal bacterial communities are of considerable functional relevance during early life. Alterations in the composition of the gut microbiota (dysbiosis) lead to immune and metabolic homeostasis dysregulation. Inadequate post-natal colonization and the consequent delay in the maturation of the intestinal barrier (gut closure) can be determined by multiple factors such as caesarean section, prolonged post-partum hospital stay, early artificial feeding, perinatal antibiotic therapy, iron supplementation and fortification [17,18], proton pump inhibitor (PPI) intake, and low APGAR score. 

Iron supplementations and iron forms adopted during post-natal period have been subject of debate in recent years due to growing evidence of the systemic long-term adverse effects caused by inappropriate iron intake during infancy [17]. Iron salts, such as ferrous sulfate, are commonly used to supplement infant formulas. Unabsorbed iron in the intestinal intraluminal environment is responsible for the dysregulation of gut microbiome composition with decreased abundance of *Bifidobacterium* spp. and *Lactobacillus* spp., which have been shown to be associated with various health benefits [19,20]. At the same time, it also favors the growth of pathogen [21,22]. The impact of supplementation with different iron salts on the intestinal microbiome and metabolome has been recently investigated by McMillen et al., who compared the effects of iron supplementation on cecal microbiome composition and metabolites in pre-weaning rat pups given oral ferrous sulfate, ferrous bis-glycinate chelate, or vehicle control [23]. Concentrations of short-chain fatty acids (such as acetate, butyrate, propionate, isovalerate, and succinate, which have pivotal roles in the regulation of immunity and metabolism), differed in a form-dependent manner compared to the control: significant differences were due to ferrous sulfate supplementation, whereas the concentrations of only two metabolite (acetate and trimethylamine) differed between ferrous bis-glycinate and control treatment groups. Standard infant formulas are usually fortified with iron in concentrations between 8–14 mg/L. In comparison, the iron concentration in breast milk is only ~0.3 mg/L, but its bioavailability is much higher compared to formulas. A 2020 Swedish randomized double-blind controlled trial (named ‘LIME study’) conducted by Björmsjö et al. demonstrated that reducing the iron content in infant formula from 8 to 2 mg/L given to a homogeneous population of healthy full-term infants does not increase the risk of iron deficiency at 4 or 6 months of age and that fortification with 2 mg/L of iron during the first six months of life is sufficient for well-nourished healthy term infants. [24]. Their findings are based on recruitments of only healthy full-term infants and indeed cannot be extended to the whole infant population. Finally, high iron intake in the post-natal period has been shown to negatively impact cognition, neurodevelopment, and brain aging in adult life [25].

As stated above, in early life, various deterministic factors seem to shape the infant gut microbial communities, including an excess of colonic iron potentially leading to the disruption of microbial homeostasis through both compositional and functional changes [26].

Compared to breast milk, standard infant formula has a lower concentration of bovine lactoferrin (or lactotransferrin, LF). LF is synthesized by exocrine glands in mammalian milk and stored in secondary granules of neutrophils, and it is a potent regulator of iron and inflammatory homeostasis due to its ability to limit iron availability to pathogens and its immunomodulatory and anti-inflammatory properties [27,28]. The Swedish LIME project investigated the immunological effects of the addition of LF (1.0 g/L) along with the reduction of iron content in infant formula on cytokines and infection-related morbidity at 4, 6, or 12 months. The authors found no relevant effects on cytokine profiles [transforming growth factor β (TGF-β)1, TGF-β2, tumor necrosis factor α (TNF-α), or interleukin2 (IL-2)] or on monitored infections (gastroenteritis, upper respiratory infections, otitis media) in infants living in a context with a low burden of infectious diseases [29]. 

Nutritional deficiencies or excesses (proteins, fibers, minerals, saturated fat, junk food) alter the microbial composition patterns, increasing the risk of developing food allergy and later other allergic phenotypes (asthma and allergic rhinitis).

Although further advances in so-called ‘omics’ disciplines are needed, microbiota biomodulators [30] (probiotics, prebiotics, symbiotics, and postbiotics) [31,32,33,34] represent an intriguing rationale in the aim of stabilizing and/or restoring a condition of eubiosis, with the associated benefits (Table 3). 

Breast milk represents the ideal food for the newborn/infant. In the case of partial or total breast milk unavailability, infant formula represents the only nutritionally adequate alternative to meet the infant’s nutritional needs. 

The major challenge in the production of infant formulas is to formulate a composition resembling breast milk as much as possible and, in turn, to reproduce the effect of breast milk on the intestinal microbiome and gut-associated immune system (GAIS) [35]. 

The effect of macronutrient differences between formulas [cow milk formula (CMF) vs. isocaloric extensive protein hydrolysate formula (EHF)] on gut microbiota has been recently examined by Mennella et al. The authors analyzed fecal samples (infants from 0 to 4.5 months randomized to receive CMF or EHF) by shotgun metagenomic sequencing and targeted metabolomics. The EHF group had faster gut microbiota maturation than the CMF group and increased alpha diversity driven by Clostridia taxa. The CMF group had faster weight-gain velocity during the first four months, greater fat mass, and higher weight for length Z-scores than the EHF group. Indeed, diet has decisive importance in shaping microbiome and modulating its functioning [36]. 

A formula should be as close as possible to breast milk, not only in terms of micro- and macro-nutrients but especially in terms of short- and long-term immune-metabolic effects. In other words, formulas should mimic as closely as possible the biological status of a healthy infant, born at term by vaginal delivery and exclusively breastfed, including the eubiotic composition of the gut microbiota. In order to achieve this, some formulas are supplemented with oligosaccharides with prebiotic action, such as fructo-oligosaccharides (FOS) and galacto-oligosaccharides (GOS) or a mixture of both, which are structurally different from maternal ones. A systematic review [37] evaluated their efficacy in healthy term-born infants fed formula supplemented with prebiotics. The review excluded trials using fermented, partially, or EHF and those supplemented with human milk oligosaccharides (HMOs). All trials recognized safety and bifidogenic effects, but only two investigated allergic manifestations. Sierra et al. reported no significant difference in atopic dermatitis, wheezing, or food allergies between the group supplemented with GOS and the control group. At the same time, Ivakhnenko and Nyankovskyy found a significant reduction in food allergies, cow’s milk protein, and atopic dermatitis in infants taking formula with GOS/FOS [38]. Still, considering that the authors of the systematic review deemed these effects to be ‘not consistent’ and that confidence intervals were wide, we can conclude that the results should be interpreted cautiously [37]. More recently, the analysis of the fecal microbial composition of infants taking formulas supplemented with GOS and FOS (BINGO and Koala studies) [39] showed similarity with the reference group (maternal breastfeeding) compared to those fed standard formula.

It is worth noting that some factors, such as maternal diet, lifestyle, infant age, delivery mode, climate, and many other environmental factors, might also have influenced the composition of infant microbiota, in addition to the effects due to different feeding modes.

A large study of more than one million Swedish children examined the association between perinatal factors (caesarean delivery, prematurity, weight for gestational age, and APGAR score) and the subsequent development of food allergies [40]. During the 13-year follow-up, the incidence of food allergy (diagnosed in a hospital setting) was found to be more frequent in females and children of mothers with asthma/lung disease, but more importantly, it was positively associated with caesarean delivery, both elective and emergency (HR, 1.21; 95% CI, 1.18–1.25). The results indicate that 17% of all food allergies could be attributable to this mode of delivery. Although this is a well-planned study, the results might be influenced by potential confounding factors such as breast-feeding, antibiotic treatments, and a lack of nutritional information. 

**Figure 2:**
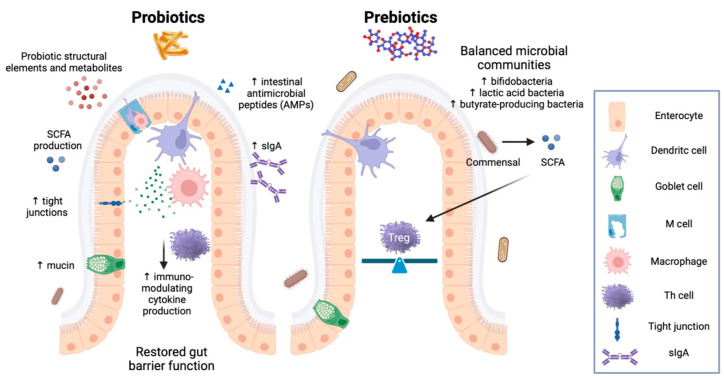
Probiotic and prebiotic mechanism of action. **Probiotics on epithelial barrier enhancement:** probiotics restore the gut barrier function by regulating the expression of genes involved in tight junction proteins (components of the apical junctional complex), by increasing the release of intestinal antimicrobial peptides (AMP) and the production of mucin, and competing with pathogenic bacteria for nutrients and colonization sites. **Probiotics on gut immune responses:** probiotics modulate pro-and anti-inflammatory cytokines or chemokines, and stimulate immunoglobulin production. **Probiotics production of short chain fatty acids:** short chain fatty acids are a subset of saturated fatty acids that include acetate, butyrate, hexanoic (caproic) acid, pentanoic (valeric) acid, and propionate. They have been shown to modulate chemotaxis; regulate cell proliferation and function; have anti-inflammatory and antimicrobial effects; and promote gut integrity. **Prebiotics** have been found to significantly modulate the balance of the intestinal microbial communities by increasing the number of lactic acid bacteria, bifidobacterial, and butyrate-producing bacteria. In addition, prebiotics could enhance host immunity by regulating immune signaling pathways and activating immune cells.

Some prebiotics and probiotic strains could mimic immunomodulation performed by an eubiotic microbiota and ensure local and systemic homeostasis by restoring the microbial compositional balance [4]. The rationale for supplementation with biomodulators is articulated through several different mechanisms of action (Table 4 and Figure 2). 

A recent systematic review evaluated the impact of probiotics (genera Lactobacillus, Bifidobacterium, Propionibacterium, Streptococcus, or mixtures), prebiotics (GOS, FOS, bovine milk oligosaccharides), and synbiotics supplementation (during pregnancy or lactation) on the intestinal microbial composition of infants born by caesarean section [41]. In the 12 eligible trials, the most used bifidobacteria were *Bifidobacterium breve*, *Bifidobacterium longum*, and *Bifidobacterium animalis*, while the use of lactobacillus strains [*Lacticaseibacillus rhamnosus* GG (LGG), *Lactobacillus acidophilus*, *Lactobacillus delbrueckii* subsp. *bulgaricus*, *Lacticaseibacillus paracasei* subsp. *paracasei*, *Lactiplantibacillus plantarum* subsp. *plantarum*, *Limosilactobacillus reuteri*, and *Lacticaseibacillus rhamnosus*] was more varied. The results confirm post-natal changes in the microbiota in these infants. The administration of biomodulators induced an increase in beneficial bacterial genera that ensure a microbial pattern more similar to that of infants born by vaginal delivery, especially regarding bifidobacteria colonization. The favorable action was most evident in breastfed babies when the intervention was early (after birth, for restoration of the bifid population) and continued after supplementation. About probiotics, the effects observed on the microbiota were most effective using multi-strain combinations.

Breast milk contains more than 200 undigestible oligosaccharides (HMOs) [42], with prebiotic, protective (pathogen anti-adhesive pathway), trophic (strengthening of barrier function junctions, production of sIgA and mucins), immune-modulating (direct pathway on immunocompetent cells and indirect via regulatory cytokines such as TGF-β and IL-10), and metabolic (short-chain fatty acids) functions. Three main categories of HMOs are generally described: neutral fucosylates (e.g., 2′fucosyl-lactose, 2′FL), neutral non-fucosylates (e.g., lacto-N-tetraose, LNT), and sialylated acids (e.g., 3′sialyl-lactose, 3′SL). Advances in biotechnology have enabled the production of certain HMOs that are added to some formulas to reduce the difference between breast milk and infant formula [43]. It is also worth mentioning that synthesized HMOs, although structurally identical to natural molecules, have limited benefits compared to breast milk. 

In any case, formulas enriched with mixtures of HMOs have been shown to support the development of the intestinal immune system and consolidate the function of the intestinal barrier through the compositional shift of the microbiota closer to that of breastfed infants (increase in bifidobacteria, especially *Bifidobacterium infantis,* and reduction in toxigenic strains of *Clostridioides difficile*).

Allergy to cow’s milk protein is one of the most common food allergies and the leading cause of anaphylaxis in childhood. Recently, its traditional management, based only on strict elimination of the offending food, has been radically changed by declining the restrictive approach and adopting a proactive one. Such a view (‘active diet therapy’) represents a valid strategy capable of facilitating the acquisition of immune tolerance.

Understanding the gut microbiome’s biological potential is paramount for innovative allergy prevention and treatment strategies. Its phenotypes, which generally disappear after developmental age, have become increasingly present in adults over the past two decades. The nutritional approach with biomodulators and a healthy dietary regimen, such as the Mediterranean diet (inscribed in the UNESCO list of Intangible Cultural Heritage of Humanity since 2010), represent indispensable preventive strategies against atopic diseases [44]. Altered microbial patterns would be involved in the multifactorial etiology of allergic epidemiological expansion [45]. The discovery of the role of nutrients in influencing the development and function of the microbiome and immune system introduced the concept of immunonutrition: epigenetic mechanisms could favorably affect the course of cow’s milk protein allergy. In the inability to breastfeed, the composition of the hypoallergenic formula could modulate these pathways. Favorable partly epigenetic effects would underlie the action of a specific probiotic strain (LGG) added to a special formula for the dietary treatment of IgE-mediated cow’s milk protein allergy. In fact, the intake of such an extensively hydrolyzed casein-based formula supplemented with LGG has been shown to reduce the incidence of other allergic manifestations and accelerate the acquisition of oral tolerance, compared to other special formulas [46]. 

Dysbiosis has also been implicated in autoimmune diseases. Although genetic predisposition and dietary exposure to gluten are considered key factors in the development of coeliac disease, alterations in the gut microbial composition may contribute to its pathogenesis [47].

## 4. Conclusions

Intestinal commensal microorganisms play a fundamental role in the regulation of immune responses. Modifying aberrant microbial patterns might counteract or mitigate the development of inflammation or allergic-related diseases. The first months of life represent a precious time window for immuno-metabolic programming, and formula supplementation with specific biomodulators is a decisive step in creating a substitute that is as close as possible to breast milk which could help to preserve the immunological and microbial compositional balance of non-breastfed infants. Although the crucial role of gut microbiota in modulating immune homeostasis is well established, the current evidence on probiotics is limited by the heterogenicity of strains, the dosage and duration of treatments, and the limited number of supplemented prebiotics (HMOs). Therefore, in order to translate this knowledge into the daily clinical practice, we need validation by both animal and large, well-designed clinical trials.

## Figures and Tables

**Table 1:** Main functions of the gut microbiota.

Function	
Protective	Countering the colonization and translocation of pathogenic and pathobiont microorganisms by the production of antimicrobial substances and competition for nutrient and receptor sites Synthesis of enzymes capable of transforming and neutralizing xenobiotic substances (e.g., drugs and, in particular, antibiotics, environmental contaminants, compounds widely used in agriculture, and zootechnics) Activation of innate and adaptive immunity Production of anti-inflammatory cytokines
Trophic Metabolic Structural	Food compound degradation Production of group B vitamins, vitamin K, biotin, and folic acid Amino acid biosynthesis Biotransformation of bile acids Regulation of fat deposits Absorption of water and minerals (iron, magnesium, calcium) Angiogenesis promotion Production of compounds with trophic function for the enterocyte (e.g., short-chain fatty acids, aminoacids, polyamines, and growth factors) Epithelial cell differentiation and growth Energy recovery Fermentation of non-digestible substrates and mucus Development of intestinal crypts and villi Reinforcement of mucosal barrier function Modulation of bone mass density Optimization of neurocognitive performance Mood modulation

**Table 2:** Human Toll-like receptors and their ligands.

Human TLR	Ligands
TLR1	Triacyl lipoproteins
TLR2	Atypical lipopolysaccharide (LPS) Glycolipids Lipoproteins Lipoteichoic acid, Zymosan Mannan Peptidoglycan Sporozoite Lipoarabinomannan Porins
TLR3	Viral double-stranded (ds)RNA
TLR4	Lipopolysaccharide Heat shock proteins
TLR5	Flagellin
TLR6	Diacyl lipoproteins Lipoteichoic acid Zymosan
TLR7	Bacterial and viral single-stranded RNA
TLR8	Bacterial and viral single-stranded RNA
TLR9	Viral and bacterial unmethylated cytosine phosphate guanine-dideoxy nucleotide (CpG) DNA RNA hybrids
TLR10	Diacyl lipoprotein Triacyl lipoprotein Viral glycoproteins Double stranded (ds) RNA

**Table 3:** Gut microbiota biomodulators.

Biomodulator	Definition
Probiotic	Live microorganisms which confer a health benefit on the host when administered in adequate amounts
Prebiotic	A substrate that is selectively utilized by host microorganisms conferring health benefits on the host
Synbiotic	A mixture comprising live microorganisms and substrate(s) selectively utilized by host microorganisms that confers health benefits on the host
Postbiotic	Preparation of inanimate microorganisms and/or their components that confers a health benefit on the host

**Table 4:** Probiotic mechanism of action.

Mechanism of Action	
Microbiological	Modulation of gut microbiota composition Competitive binding to intestinal receptors (prevention of pathogen invasion) Bacteriocin production (preventing the growth of pathogens)
Structural	Modulation of epithelial barrier function Production of short-chain fatty acids (strengthening of the intestinal barrier with anti-inflammatory action) Regulation of tight junction protein expression (reduced antigen transfer)
Immunologic	Probiotics play a role in host innate and adaptive immune responses by modulating immune cells such as dendritic cells (DCs), macrophages, and B and T lymphocytes Modulation of Th1/Th2 lymphocyte ratio Activation of T regulatory (Treg) cells Maturation of B cells into immunoglobulin (Ig)A-producing plasma cells.

## Data Availability

Not Applicable.
